# *QuickStats:* Percentage* of Adults Aged ≥18 Years Who Felt Very Tired or Exhausted Most Days or Every Day in the Past 3 Months,† by Sex and Age Group — National Health Interview Survey,^§^ United States, 2022

**DOI:** 10.15585/mmwr.mm7245a7

**Published:** 2023-11-10

**Figure:**
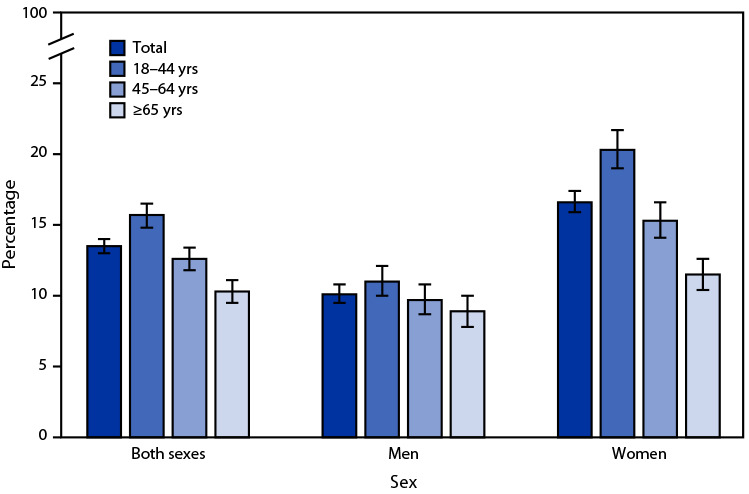
In 2022, 13.5% of adults aged ≥18 years felt very tired or exhausted most days or every day in the past 3 months, and this percentage declined with age. Among men, the percentage was highest among those aged 18–44 years (11.0%), followed by those aged 45–64 years (9.7%) and ≥65 years (8.9%). Among women, the decline in the percentage with age was steeper, decreasing from 20.3% (18–44 years), to 15.3% (45–64 years), to 11.5% (≥65 years). The percentage of adults who felt tired or exhausted most days or every day was higher for women compared with men in each age group.

**Source:** National Center for Health Statistics, National Health Interview Survey, 2022. https://www.cdc.gov/nchs/nhis/index.htm

